# Integrated visual analysis of protein structures, sequences, and feature data

**DOI:** 10.1186/1471-2105-16-S11-S7

**Published:** 2015-08-13

**Authors:** Christian Stolte, Kenneth S Sabir, Julian Heinrich, Christopher J Hammang, Andrea Schafferhans, Seán I O'Donoghue

**Affiliations:** 1 CSIRO, Sydney, Australia; 2 The Garvan Institute of Medical Research, Sydney, Australia; 3 The University of Sydney, Sydney, Australia; 4 Technische Universität München, Munich, Germany

**Keywords:** Molecular Sequence Analysis, Molecular Structural Biology, Computational Proteomics

## Abstract

**Background:**

To understand the molecular mechanisms that give rise to a protein's function, biologists often need to (i) find and access all related atomic-resolution 3D structures, and (ii) map sequence-based features (e.g., domains, single-nucleotide polymorphisms, post-translational modifications) onto these structures.

**Results:**

To streamline these processes we recently developed Aquaria, a resource offering unprecedented access to protein structure information based on an all-against-all comparison of SwissProt and PDB sequences. In this work, we provide a requirements analysis for several frequently occuring tasks in molecular biology and describe how design choices in Aquaria meet these requirements. Finally, we show how the interface can be used to explore features of a protein and gain biologically meaningful insights in two case studies conducted by domain experts.

**Conclusions:**

The user interface design of Aquaria enables biologists to gain unprecedented access to molecular structures and simplifies the generation of insight. The tasks involved in mapping sequence features onto structures can be conducted easier and faster using Aquaria.

## Background

The number of protein sequences collected in public databases such as UniProt [1] has been growing exponentially over the last decade, and will continue to grow even faster with the advance of sequencing technologies. Currently, UniProt listed more than forty million protein sequence entries. In fact, the total number of known protein sequences is substantially larger, since individual UniProt entries typically document multiple sequence variants deriving either from single-nucleotide polymorphisms (SNPs) or from splicing.

Proteins are involved in nearly every biological process and can be viewed as the molecular machinery of life. To fully understand the biological functions of a protein, however, life scientists need to know much more than just its amino acid sequence -- one very rich source of additional knowledge are the three-dimensional (3D) structures (unless otherwise stated, in this work we will use the term *structure *to refer to atomic-resolution three-dimensional models of protein molecules, or of proteins in complex with other macromolecules) adopted by a protein across a range of physiologically relevant conditions. Where available, such structures can give detailed insight into the molecular mechanisms underlying a protein's function. Unfortunately, the experimental determination of protein structures lags significantly behind sequencing; currently, the protein data bank (PDB) [2] holds slightly more than 100, 000 structures, thus comprising less than 1% of the available sequences in UniProt. Due to this paucity of structural information, many of the widely-used genome analysis pipelines overlook protein structures.

However, for many of the > 99% of proteins that lack any experimentally determined structures, some structure information can usually be inferred via *homology modelling *or *comparative modelling *methods [3]. These methods take advantage of the well-established observation that proteins with similar sequences tend to have similar structure [4-6]. For the remaining proteins with no detectable sequence similarity to proteins of known structure, methods have been developed that attempt to predict structure *ab initio *from amino acid sequence -- sometimes considered as the "holy grail" in bioinformatics [7]. When using structural models derived from these methods, scientists need to be very aware that the models contain regions with variable levels of uncertainty, and will often contain considerable inaccuracies, especially *ab initio *models. The quality of a model depends on details of the often very complex method used to derive it -- it can be difficult to communicate this information clearly to end-users unfamiliar with modelling methodologies, with the very real danger that incorrect conclusions may be drawn by inexpert users.

Avoiding such misinterpretations was a key motivation behind the development of a related approach taken by the SRS 3D system [8], which was recently superseded by the Aquaria resource [9,10] developed in our laboratory. Instead of calculating model structures, the Aquaria approach calculates sequence alignments between all known protein sequences and all known protein structures [11] (using a so-called 'HMM-HMM' search strategy [12]), and displays experimentally-determined structures overlaid with abstract data to indicate the quality of the sequence match [8,9]. This approach also makes it possible to visualise any sequence-based *feature *or *annotation *such as SNPs or post-translational modifications (PTMs) in their spatial context, which often helps in understanding of the effects of such features on a protein's function.

There are many approaches to query for and visualise protein molecular structures, many accessible from the PDB [2,3]; only some methods provide access to the amino acid sequence related to each protein structure, however most provide either no interactive connection between sequences and structures or require multiple steps to switch between both representations, impeding the process required for the user to construct mental models of the problem at hand. Only a few tools provide linked 3D structure and sequence visualisations (e.g., Cn3D [13], STRAP [14], and UCSF Chimera [15,16]), however they do not scale well for the visualisation of large numbers of sequences, as amino acid sequences are rendered using one-letter codes exclusively and thus provide only little or no aggregation with respect to sequence length and alignment size. Aquaria is unique in the degree to which its user interface is organised primarily based on sequence, not structure; it is also unique in providing a degree of accessibility and scalability that makes it feasible for users to visually analyse a large number of protein sequences and structures.

The Protein Model Portal [3] systematically computes structural models from protein sequences using various comparative modelling algorithms. In contrast to Aquaria, each model structure presented in PMP can be derived from multiple experimental structures, is not verified experimentally, and contains uncertainties and sometimes inaccuracies that may not be easily understood by the end user. The result of a query is presented as a list of matching models and a corresponding sequence alignment, but in order to see a structure, multiple links have to be clicked by the user. The PDB provides experimentally determined structures and offers a rich set of information for each query, including an interactive structure viewer, but without interaction to the query sequence and associated features. Finally, there is a wealth of websites and applications both open-source and commercial that provide visualisation for sequence alignment and analysis, but without linking to structures [17].

Although the models presented in Aquaria are not as refined as those derived from modelling [3], the uncertainties and inaccuracies can be much more easily understood by molecular biologists and biochemists who are not expert in structures or homology modelling -- this is aligned with our goal in creating Aquaria, which was to make structural informational more accessible to a much broader community. Aquaria has been quite successful already: in the first three months after it was launched (in February 2015), the resource has attracted more than 6,000 users, who spent an average of 4.2 minutes each on the site.

While the Aquaria resource and the details of the underlying database are described in detail elsewhere [9], this paper focuses on the design decisions that were made for its visual interface. In particular, we present (i) a problem domain characterisation and overview of tasks biologists frequently need to conduct in order gain insight into protein structures, a set of (ii) design decisions made for a visual query interface to support these tasks, and (iii) two case studies demonstrating the use of the system.

## Methods

The development of the Aquaria user interface was an iterative, user-centred design process, based upon understanding of users, tasks, and context. Visual mock-ups were created in Adobe Illustrator and Adobe Photoshop for each design phase and for each state of interactive user interfaces. Colours were soft-proofed in Photoshop for the two most common types of colour blindness, protanopia (reduced sensitivity to red) and deuteranopia (reduced sensitivity to green). Users of different levels of expertise, skills, and experience were involved throughout design and development, and the design was driven and defined by user-centred evaluation. Here, we describe the requirements and design decisions as a result of this process.

### Requirements analysis

In this section, we provide a requirements analysis for designing a visual interface to the database underlying Aquaria. This includes a characterisation of the problem domain and a list of common questions biologists seek to answer in order to gain insight from protein structures.

Structural biologists can spend months and years working on a single structure, and hence are often very knowledgable about structures for a relatively small set of proteins; in contrast, most other biologists and molecular biologists require much less frequent access to structural data, and, when they do view structures, will usually ask less specialised questions. Hence, Aquaria is targeted mainly at life scientists who do not have expert knowledge about the structural biology of a particular protein. These target users are typically interested in first gaining an initial overview of which related structures are available for a given protein, then selecting which structures are most relevant to the biological phenomena they are most interested in understanding.

To understand more precisely which key questions biologists in this situation will need to address, we conducted detailed discussions with two structural biologists, a cell biologist, a chemist, and two bioinformaticians - they showed us how they currently answer these with existing systems, and provided us with questions they are frequently facing (Table 1). This list of questions was further validated and refined based on informal discussions with a large number of biologists, following numerous seminars and presentations over a four-year period in which the questions were presented.

**Table 1:** Common questions about protein structures biologists seek to answer and the visual encoding and/or panel used in the Aquaria user interface to address these.

**No**.	Question	Visual Encoding and/or Panel
1	For a given protein sequence, how many related 3D structures are known?	Matching Structures (= MS)
2	Do any structures match exactly? If not, what is the best match?	vertical position & colour-code (MS)
3	Which structure spans most of the given sequence?	horizontal position (MS)
4	Which molecular states occur amongst all related structures?	thumbnails (MS)
5	For a given domain, how many related structures are known?	horizontal position (MS)
6	For a given domain, do any structures match exactly? If not, what is the best match?	position & colour-code (MS)
7	For a given domain, which molecular configurations occur in the related structures?	tree level 1 (MS)
8	For a given domain and molecular configuration, which PDB entries are related?	tree level 2 (MS)
9	In a large molecular assembly, where is a given protein? How does it interact with others?	semi-transparency (3D Structure)
10	How well does a structure match to a given protein sequence?	colour-code (3D Structure)
11	Which residues in the structure differ from the specified protein sequence?	colour-code (3D Structure)
12	Which residues in the structure differ in a related organism (e.g., in mouse)?	colour-code (3D Structure)
13	For a given residue in the structure, where is it in the sequence?	3D Structure
14	For a given residue in the sequence, where is it in the structure?	3D Structure
15	Where in the structure is the N-terminus? Where is the C-terminus?	3D Structure
16	Which residues make contact with ligands? Or with other proteins?	3D Structure
17	Which kinds of features are available, and how many?	Features
18	For a given feature (e.g., a domain, PTM, or SNP), where is it in the sequence?	Features
19	For a given feature (e.g., a domain, PTM, or SNP), where is it in the structure?	Features & 3D Structure
20	For a given structure, where are a set of features located (e.g., all domains, PTMs, or SNPs)?	Features & 3D Structure
21	For each partner of a given protein, ... [repeat questions 1-20]?	3D Structure

While we found that most of these questions can be addressed using a combination of the existing systems and public databases described in the previous section, doing so can be difficult or even impossible due to a lack of integration of the required resources. One important issue we found in using the existing tools to address the questions listed in Table 1 is that a user is forced into many context switches, as information about structures, sequences, and features needs to be gathered from different sources throughout the web. This often includes the translation and verification of identifiers from one database to another, e.g. to retrieve an interactive view of a structure given a list of entries in the protein model portal. Switching to a spatial representation of a sequence feature now typically requires the user to mentally project these features onto the currently active 3D representation.

### Design decisions

In this section, we describe the design decisions made for the Aquaria user interface to help biologists answer the questions listed in Table 1.

Since the primary design goal was to create an accessible and easy-to-use interface to protein structures for a wide range of users, the first design decision we made was to build our tool as a web-based resource, for two reasons: first, to facilitate easy access to the databases which contain related information about protein structure and features (including UniProt, Swiss-Prot, PDB, InterPro [18], SignalP [19], and Pride [20]), and second, to make the tool broadly and easily accessible via a web-browser.

Our overall approach was to adhere to the visual information seeking mantra [21] by providing overviews that scale with the number of results produced by the system as well as details on demand. This is achieved by applying hierarchical aggregation techniques for sequence information and focus+context techniques for 3D structural views.

**Figure 1:**
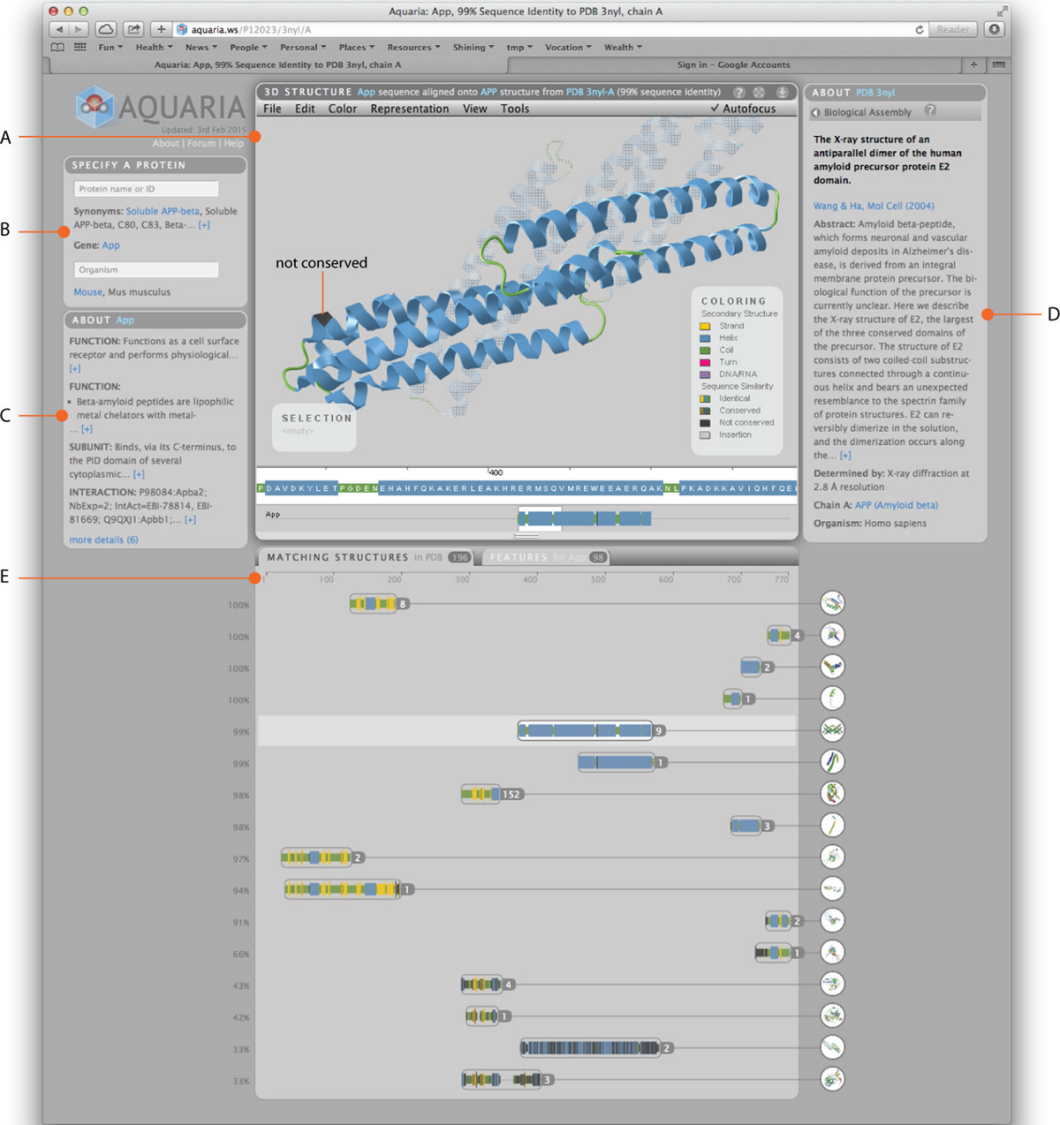
**The Aquaria user interface comprises five panels**: the 3D structure panel (A) shows the currently selected 3D structure with various rendering modes using the same colour scheme as applied for all structures and aggregates (E) that match a given user query entered through the search panel (B). A white background is used to visually connect the sequence being rendered in the 3D view and its cluster in the matching structures panel (E). Panels on either side give information about the Uniprot entry corresponding to the query (C) as well as details of the structure (D) being shown in the 3D structure panel.

The user interface of Aquaria is divided into multiple *panels*, each of which is conveying different information about the user query (Figure 1). First, we briefly describe the overall layout and purpose of the panels, followed by a brief section detailing the contents and purpose of each panel with regards to the questions in Table 1. Finally, we describe some of the design decisions that affect multiple panels.

#### Panel layout

The *search panel *(Figure 1B) comprises of two fields: one for the protein of interest, the other for organism name. To distinguish the protein of interest from other protein sequences and structures, we will refer to the protein that was queried for as the *specified protein *for the remainder of this article. The search panel is located in the top left because searching for the specified protein is the first operation required by the user.

As protein structures play a key role in answering the questions listed in Table 1, we decided to put the *3D structure panel *at the centre of the user interface (Figure 1A). At this position, the attention of a user is guided to the most similar of all available matching structures, which is selected by default after entering a query and enables a very quick initial assessment of how the specified protein's structure might look like.

Below the 3D structure panel in the centre is a panel with two tabs: *Matching Structures *and *Features *(Figure 1E). This panel is located at the bottom of the page to allow for vertical scrolling through results, as for some proteins, there may be thousands of matching structures, and hundreds of annotated features.

Below the search panel resides the *information panel *about the query protein (Figure 1C), taken from information in UniProt. Since text for certain details is often very long, it is abbreviated to three lines by default, but easy to expand on demand.

The information panel on the right shows facts *about the structure *(Figure 1D), which are derived from the PDB.

#### Search panel

Both the protein and organism search fields support autocomplete to give users instant feedback about the contents of Aquaria's database. As protein sequences (as required for all questions in Table 1) can be referred to via the sequence itself, the corresponding gene, various identifiers from different databases, or the actual PDB id, the query may contain any of these terms. This frees our users from the time-consuming and error-prone task of translating identifiers between these databases. The query results are grouped by source title within the autocomplete dropdown to avoid confusion between similar looking identifiers from different sources.

#### 3D structure panel

The structure panel used in Aquaria evolved from the one that was developed for SRS3D and includes an interactive 3D viewer for molecules with support for the most common rendering modes as well as a sequence view showing the sequence of the current structure. Here we describe some of the improvements we made to adapt this panel to Aquaria.

Most noticeable is the choice of grey as background colour: grey reduces contrast to avoid eye strain, and provides best visibility for all residues, since it allows for highlights and alignment gaps, rendered in white, and non-conserved residues, rendered in black, to stand out. For printing and export of images, we added optional white or black backgrounds.

The relationship between the sequence view and the structure view has potential for confusion, since the sequence view only shows a single protein chain, while a single PDB structure may be comprised of multiple similar or different protein sequence chains. To alleviate this discrepancy, we implemented 'autofocus': the Aquaria 3D viewer makes the other non-related chains in the PDB structure semi-transparent, while the selected chain is completely opaque. This supports the user in answering Question 9, as for large assemblies (comprising multiple protein chains), fully opaque structures would hinder the view on the chain associated with the specified protein. 'Autofocus' can be disabled via the menu bar at the top of the 3D view.

If the user selects one of the semi-transparent residues in the other chains, then the chain of the selected residue will become opaque and the centre of rotation, as it is now the selected chain, and the pre-existing selected chain is made transparent. In order to allow the user to explore the binding partners of a molecule (cf. Question 21), the specified protein will be replaced by the sequence of the newly selected chain and all other panels will be updated accordingly.

The SRS3D viewer supported a number of feature annotations that would be displayed below the 3D model as selectable rows, and the features would be mapped to the 3D object when selected by the user. However, nowadays annotations for a protein may number in the thousands, which would not scale well when displayed within the view frame. Aquaria limits the number of features that can be loaded into the view to one and provides a separate interface for navigating and selecting features from a list to be sent to the view.

#### Matching structures panel

Questions 1 to 8 in Table 1 seek answers to the relation of structures that are available for the specified protein. To accomodate for the associated tasks of visually assessing the range (Questions 1, 3, 5) and quality (Questions 2, 6) of structural matches onto the specified sequence, we decided to use a design that resembles an alignment view of structures on the sequence of interest (Figure 1E).

However, structural matches as reported by the Aquaria database range from several dozen to thousands of results for a specified protein. While the position of the Matching Structures panel at the bottom of the layout provides the option to expand the panel to fit any amount of content, having to scroll too much would be an obvious impairment of the workflow. Hence, we used aggregation on two levels to get a good trade-off between the overview required for Questions 1 to 6 and the details required to answer Questions 7 and 8:

At the sequence level, functional elements occupy one or more positions, defined by start- and endpoints. Search results are aggregated at the first level by position in the alignment. Within each group, sequences are ordered by identity relative to the query sequence, whereas the visual representative is the top-ranked structure within each group. Next to the sequence image, which shows the secondary structure and indicates the degree of similarity with the query sequence, we placed a numeric label stating the size of the group. To the right side of the panel, a thumbnail of the structure image from PDB provides useful information for those familiar with proteins. This allows one to visually assess the quantity, quality, and position of the best structural matches for the specified protein.

At the next level of aggregation, sequences are grouped by molecular configuration (Questions 7 and 8): conformation (monomer, dimer, etc.), symmetry (homo-dimer vs. hetero-dimer, etc.), and binding partners the structure includes. Within this grouping, structures are ordered by identity and crystallographic resolution, with NMR structures last.

To navigate inside the group, a click on the numeric label opens a collapsible tree structure, showing the next level of grouping. For large groups, this poses the problem how to manage the display of potentially thousands of nodes. We employed a paging-based strategy:

The tree is drawn, centred vertically on the first-level group, limiting the number of nodes to what can comfortably fit in the available space. We use pagination to accomodate for larger sets of nodes.

To avoid spacing problems arising from the position of nodes expanded inside the tree, all nodes in the level undergoing selection are collapsed and the chosen node moves to the centre, while the next level of the tree is expanded. This results in a straight line of parent nodes, which can also be navigated in reverse: when clicked, the child nodes collapse and the nodes on the parent level are made accessible again.

On the second level of aggregation, again, each node has a numeric label indicating how many structures it contains, which acts as the trigger to show the next level of the tree. This third level is where the user can select a structure to load into the 3D view by clicking on the thumbnail image.

#### Features panel

Sequence-based *features *or *annotations *are a rich source of information available on the web. These features, however, can not be easily mapped onto a spatial context, as they naturally occur in proteins. Questions 17 to 20 are about visualising features on one of the matching structures.

**Figure 2:**
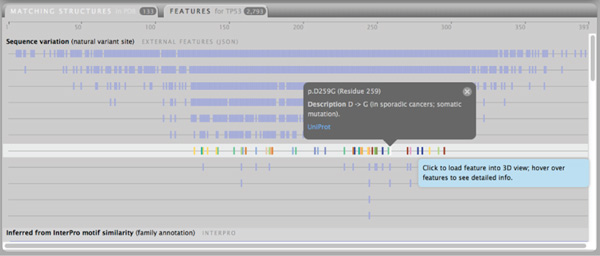
**Sequence variation features for P53**. The features panel shows a collection of annotated tracks derived from various sources that can be used to map features onto the 3D structure displayed in the 3D viewer. Here we show a large number of natural variant sites (mutations) for the tumor suppressor protein P53. Overlapping features are drawn in separate lanes, thus enabling users to identify the residues that have largest number of distinct mutations, and map these onto structures.

**Figure 3:**
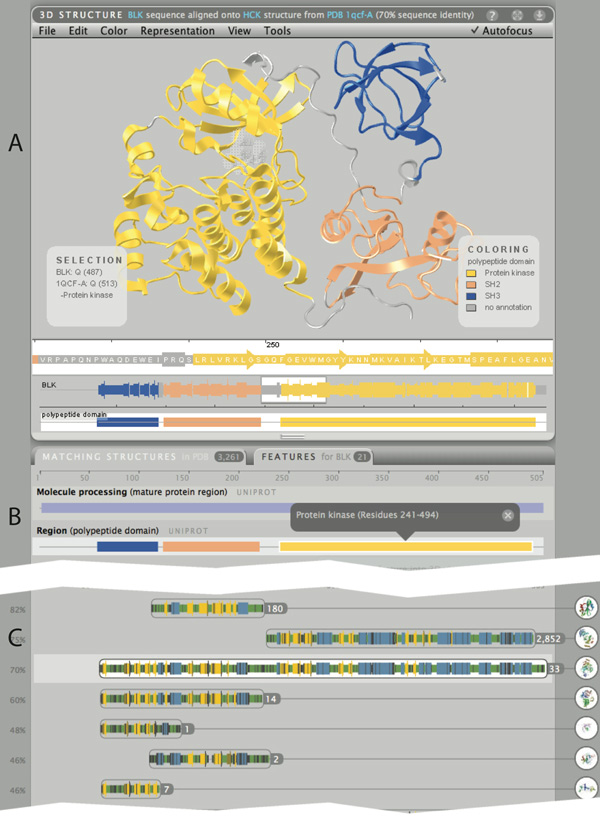
**Structures and features for human BLK protein**. (A) BLK contains three independently evolved sequence domains, each with its own function. By mapping the UniProt "domain" feature set (B) onto the structure automatically selected by Aquaria, the user is able to clearly see which parts of the structure correspond to which domain. (C) The Matching Structure panel shows that there is no PDB structure exactly matching the BLK sequence, while many (over 3,000) related structures exist, thus providing a wealth of detail on the molecular processes of related proteins. Most of the related structures match to the kinase domain; many match to either the SH2 or SH3 domain, while a small number contain all three domains in the same order as BLK.

**Figure 4:**
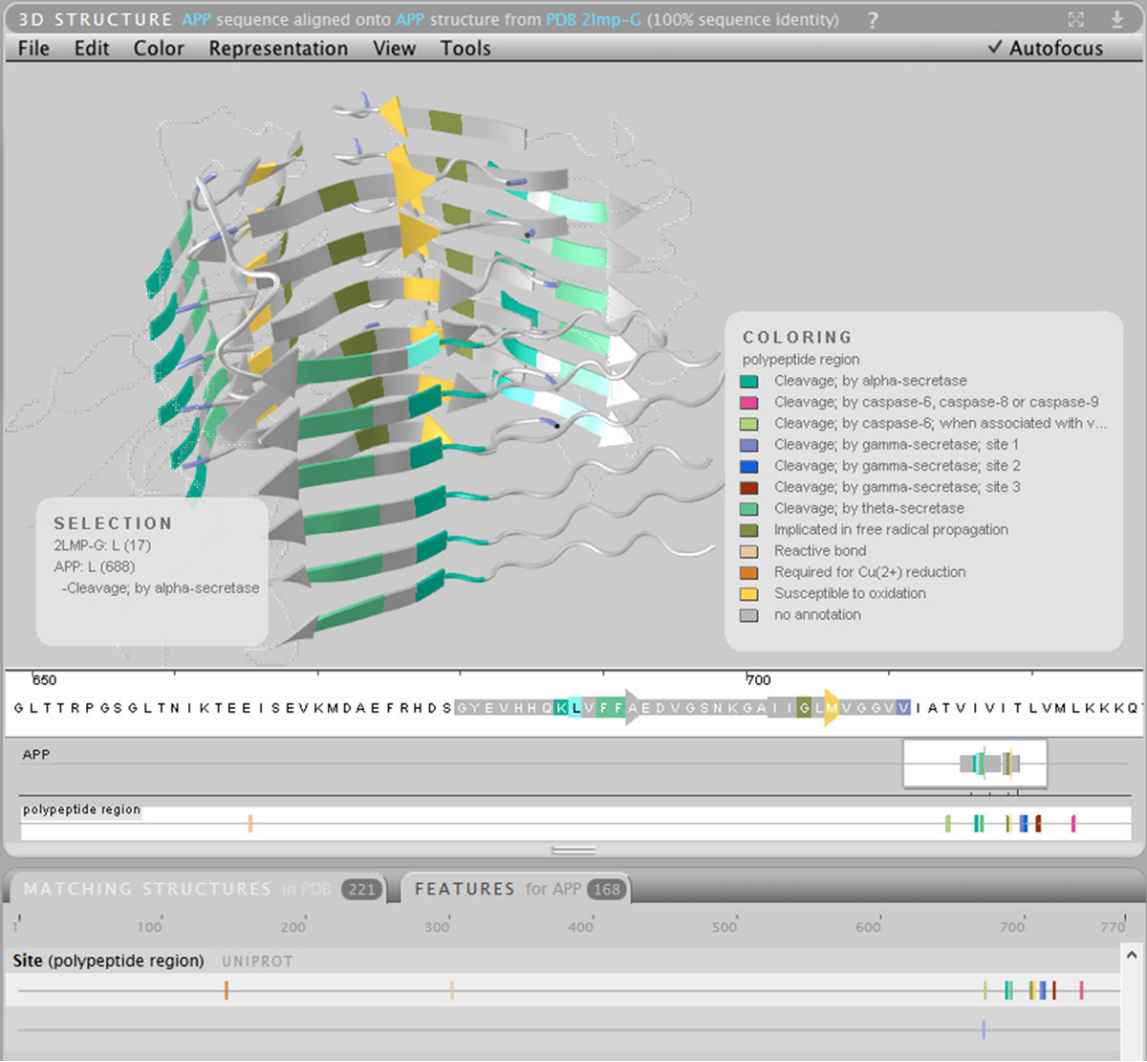
**Atomic structure of an amyloid fibre formed from APP**. The structure (PDB 2yti) has been mapped with the UniProt "polypeptide region" feature lane, which indicates the location of enzymatic cleavage sites. If the indicated cleavages by gamma secretase (at residue 711) or alpha secretase (at residues 687-688) occur, the APP amyloid fibre could not form. Thus, this figure shows the power of combining features with structures, as it suggests potential research directions that could be pursued as potential treatments for Alzheimer's.

The features panel (Figures 2, 3, and 4) shows annotated features collated from a variety of resources, thus making it easy for the user to access most publicly available information without leaving the Aquaria user interface. We group features of the same kind from each resource into one 'track', which results in a compact display of features for most proteins.

One phenomenon of protein features is that there can be multiple annotations for the same region of a protein sequence. For single-residue features, such as mutations, one option is to display feature density, which uses less space by displaying all features of one category in one track. For multi-residue features, overlaps could be made visible by employing transparency. Both approaches, however, create ambiguity when trying to use them in an interactive context: when hovering over an area with overlapping features, meta-information for many instances would need to be displayed at the same time. This kind of information includes additional information about a single feature occurring at a particular position of the specified sequence.

Instead, we chose to draw overlapping features in separate *lanes*, which give users access to metadata for each feature. For feature types with a large number of annotations, as highly-studied proteins often have, this method produces a histogram-like display with the most frequently varied residues shown in the lower lanes (see Figure 2).

Initially, all annotations are displayed in uniform colour to give the user a clear overview of the distribution of features. When hovering over a feature lane, colours that set apart different features become visible; when hovering over any individual feature, metadata about its nature, position, and a link to the resource of origin are displayed.

When clicked, features from the chosen lane are loaded into the 3D view, colouring the currently displayed structure according to their position. In addition, a feature track is created below the sequence view in the 3D structures panel. In the features panel, the active feature lane is marked with a lighter background and remains coloured until it is clicked again, which unloads the feature from the 3D view and removes the feature track from the sequence view.

#### Sequence and structure representation

Sequences are displayed using the same visual encoding in several locations in the user interface, but at different scales: at the bottom of the 3D view, a sequence track at the single-residue scale is shown, with the view centred on the residues that are currently selected in the structure. Each residue contains a one-letter code to indicate amino acid types, rendered using a fixed-width typeface. Below this detail view we provide an overview track with a sliding window that indicates the overall position of the highlighted sequence. In this display, residue width is scaled down to show the alignment position of the displayed structure against the specified protein sequence, which is drawn as a thin, dark line spanning the entire width of the panel. The same sequence is also displayed in the Matching Structures panel, along with that of other matching structures. Again, residue width is scaled to show the alignment position of each structure against the full length of the query protein sequence, represented by the ruler at the top of the panel. To visually connect these sequence representations at different scales, we usa a white background rendering for the selected parts of a sequence in the 3D view and the Matching Structures panel (cf. Figure 1).

Proteins can be organised into secondary structure elements: strand, helix, coil, and turn. The 'cartoon' rendering style is based on these elements and thus provides visual cues or 'landmarks' which supports orientation. We reinforce this by employing a consistent colouring scheme for secondary structure throughout the Aquaria user interface: strands are yellow, helices blue, and coils green. To represent these landmarks in a sequence context we decided to use both colour and shape, a strategy which provides safety through redundancy: solely employing height variation and shape to represent secondary structure elements may be ineffective when these shapes are compressed to fit the screen width, which often happens for large proteins. Even in situations where single residues are scaled down to one pixel or less, regions with differences are recognisable due to changes in colour.

For the shapes to represent secondary structure in a sequence context, the goal was to reduce cognitive load by finding a visual vocabulary that was closely related to the default ribbon-style rendering used in the 3D view. Here, helix elements are rendered as corkscrew-like ribbons, strands are flat ribbons with arrowheads, and coils are thin, spaghetti-like shapes following an irregular path.

Other requirements were dictated by the fact that we wanted to display sequences aligned to each other: residues all needed to be rendered at the same width. This required a modular system, where edges of the various elements had to be parallel in order to construct consecutive regions of secondary structure elements. Lastly, the elements needed to be of sufficient height to accommodate uppercase letters, as is the case for the sequence detail view.

Unlike genomic sequence, the basic unit for protein sequence is an amino acid, referred to as "residue". With twenty possible residue types, substitution matrices become very complex; for the purpose of displaying differences resulting from the alignment of two protein sequences, we decided to assign residues to one of four categories with the following attributes:

• *Identical*, displayed in the original colour of the secondary structure for that residue

• *Conserved *(for chemically similar substitutions), shown diminished in saturation and brightness

• *Not conserved *(for substantially different substitutions), shown in dark grey

• *Insertion*, shown in light grey

The resulting display of structures allows users to judge the quality of a structure model intuitively by its chromatic quality: the less saturated and bright the colours in a structure appear, the less similar its sequence is to that of the query protein. This effect is particularly noticeable in the Matching Structures panel, where structures are ordered by percent identity, with the highest-ranked structures displayed at the top.

However, in order not to rely on colour coding alone, we also display a precise percentage of identity for each structure (in the title bar of the 3D viewer, and to the left of each group in the Matching Structures panel).

## Results

In this section, we present two case studies that demonstrate the effectiveness of Aquaria for using structures to learn about protein function.

### B-lymphocyte tyrosine kinase (BLK)

For this case study, we focused on BLK, a tyrosine kinase involved in B-lymphocyte development, differentiation and signaling (Uniprot id P51451). To gain information about a protein in Aquaria, the first step is typically to query using the protein name, synonym, or primary accession. In this example, the user first needs to confirm that the organism is set to "Human", then enters "BLK" into the query field. A list of 3, 261 matching structures is displayed -- a wealth of structural data that can provide insight into the molecular mechanisms occurring in related structures. These structures are clustered by homology into 33 groups through finding all sequences of structures that match a particular range of the given protein (Figure 3 shows the initial view after entering the query). This simplified view provides the user with an initial visual assessment of the diversity of experimentally determined structures that are available for the query. For BLK, it shows multiple overlapping clusters (Figure 3C) that give rise to the assumption that this structure is composed of multiple domains, each matching with a variety of structures in the PDB.

The structure that is displayed initially in the 3D viewer is from the third cluster; while this structure has has a lower sequence identity to BLK that structures in the top two clusters (70% compared with 82 and 75%), it has a large total number of identical residues, when aligned onto the full-length BLK sequence. The width of each cluster shows the length of the alignment, while the color is used to communicate the quality of the top ranked structure in each cluster. Clicking on a cluster group loads the best matched protein structure of that cluster into the 3D viewer. When a new structure is loaded, the text on the right -- containing bibliographic information about the structure -- is updated accordingly. The default selected cluster contains 30 similar PDB structures based on homology matching for that group. By clicking on any of the clusters, a tree appears showing the respective PDB structures grouped by macromolecular assembly and binding partners into sub-clusters (Figure 3). Drilling down to a dimer and selecting one of the entries displays the respective PDB structure in the 3D viewer. As this is a dimer, one of the chains is fully opaque, centred and is the centre of rotation while the other chain is semi-transparent.

The features tab shows 17 features for BLK protein, including Uniprot and Interpro domains, binding motifs, post translational modifications, amino acid modifications, single nucleotide polymorphisms (SNPs), and other experimental information. Clicking on the top feature loads the feature track into the 3D viewer highlighting the different domains in the 3D structure (see Figure 3A, bottom).

In summary, the wealth of structural information in the matching structures panel show the power of Aquaria in delivering structural insight for BLK, even though there are no exact matches in the PDB. The views of the BLK structure enhanced by mapping domains and other features proved very insightful, as they allow users to understand which parts of the protein perform the functional roles associated with each domain.

### Amyloid precursor protein (APP)

For this study, we examined the process used in creating a 3D model representation of the overall structure of amyloid precursor protein (APP), a molecule which is considered important in the development of Alzheimer's Disease. This model was designed to be used in an animated visualisation which explores the process of Alzheimer's disease associated neural plaque formation, and therefore was intended for a general audience. The work was conducted by one of the authors (CJH), a biomedical animator trained in medical science.

The elements which were to be visualised included the overall structure of the APP protein, its localisation within the cell membrane and the enzymatic degradation of the protein.

An investigation into the structure of APP was conducted in order to build a model which was consistent with the up-to-date understanding of the known structure of the protein. Aquaria shows that no single structure spanning the entire APP protein has yet been resolved (Figure 1), however a range of structures have been determined covering different domains along the protein, thus it is a suitable use case for Aquaria.

The modelling process involved using Aquaria to assemble the relevant data from the protein data bank (PDB), reading a series of reviews into the overall structure and cellular distribution of the APP protein and piecing together PDB structures using Blender and the embedded python molecular viewer (ePMV) to create the model and animate it for a final video.

We searched Aquaria for related structures by using the keyword "APP" and selecting "human" as the organism. Aquaria's top match result is UniProt entry P05067 which was the appropriate result in this case (Figure 1).

In Aquaria this identifier currently yields 221 structures which span most of the full sequence (Table 1, Q1). Most of the regions which have sequences for APP have a structure with 100% identity to the search query (Table 1, Q2 and Q5). The largest structure is present in the E2 domain, a coiled coil structure which spans 195 residues (Table 1, Q3). This structure contains a dimeric assembly; Aquaria helps in interpreting the structure by initially highlighting just one chain (Table 1, Q7 and Q9), thus simplifying the view. One of the domains (the "Kunitz protease inhibitor domain") has over 150 matching structures in Aquaria (Table 1, Q5).

Using the features tab for aquaria reveals several features of the protein:

• It is a transmembrane protein as seen in the features view for Region "Transmembrane", with residues 700-723 occupying the membrane (Table 1, Q18-20).

• The majority of the protein is in the extracellular region (681 of 770 residues) on the N-terminal end (Table 1, Q18-20).

• Mapping the Region "polypeptide region" onto structure 2lmp gave insight into the precise location of enzyme activity on APP via alpha and gamma secretase, which are known to cleave APP (Table 1, Q13). Alpha secretase cuts the protein at positions 687-688. There are 3 sites for gamma secretase activity, all within a small region of the intermembrane region. Beta secretase activity occurs at residues 671-672 (Figure 4).

• Examining the molecule processing feature lane titled "Mature Protein Region" gave insight into the precise location of enzymatic cleavages that occur within and flanking the intermembrane domain (Table 1, Q13).

**Figure 5:**
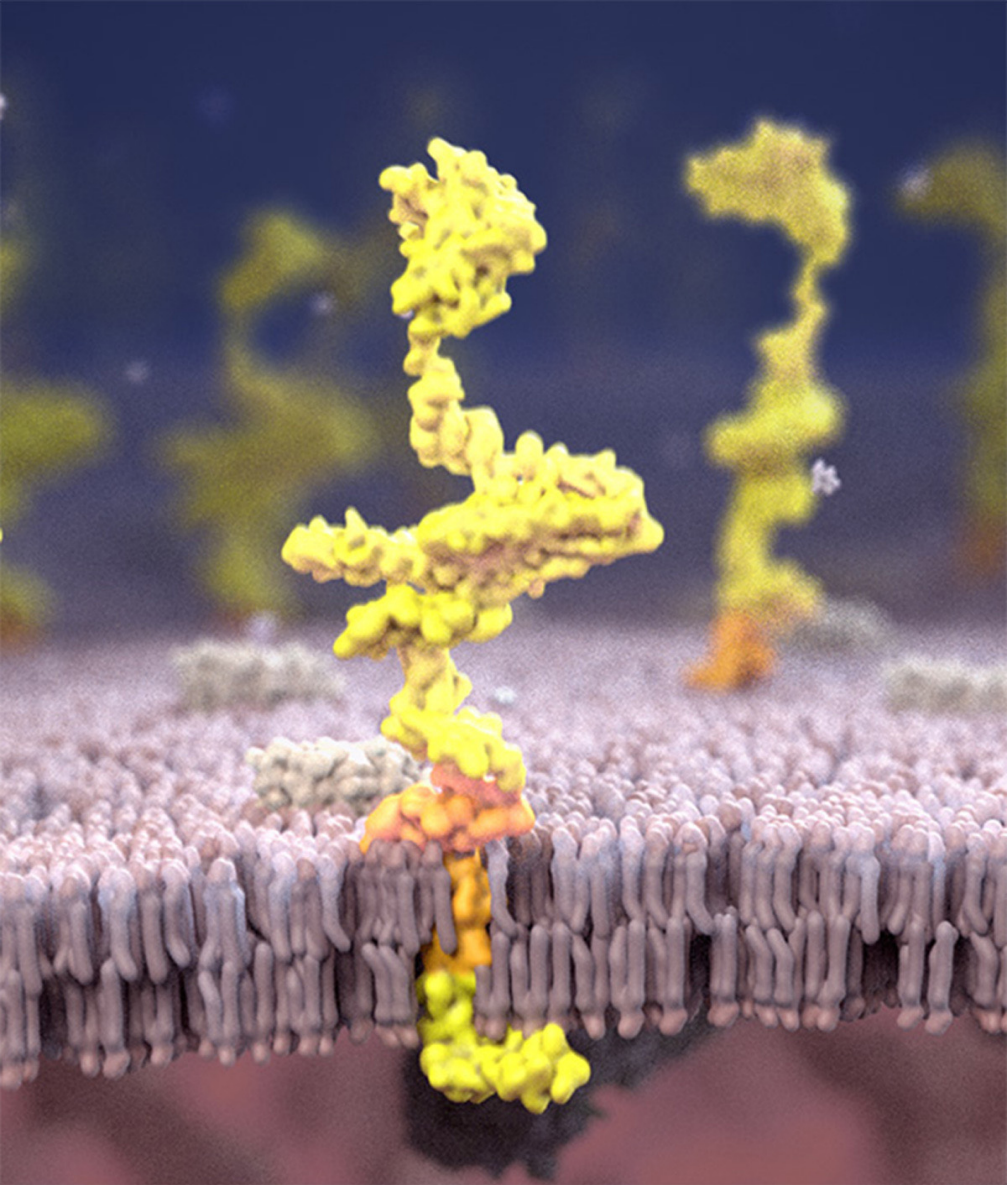
**Screen capture from APP animation**. This shows the final model for the overall structure of APP assembled using Aquaria (yellow). The protein is enzymatically cleaved at several positions; the region of the protein that eventually forms part of the amyloid fibre is shown in orange.

From the Aquaria interface, by tracking the literature for each structure observed, we found a recent literature review that discussed the current state of knowledge regarding the overall structure and subcellular location of APP [22]. This gave additional insights into the structure that were not determined from Aquaria itself; Isoform APP695 is most prevalent in the nucleus (this variant lacks the kunitz protease inhibitor domain). Sequence alignments in the paper demonstrate that evolutionarily conserved regions exist in the protein. The insights gained from Aquaria and subsequent literature analyses were then used to construct an integrated 3D model of APP in Blender (Figure 5); the model was then used to create an educational animation [23].

The key benefits we found using Aquaria for this case study were:

• Clarity as to which regions of the protein did and did not have resolved structures

• Access to an organised and coherent assembly of all PDB entries relevant to the protein of interest

• Clear insight into the subcellular location of APP, and into which residues contact interaction partners

• An indication of the integrity and match identity of the available structures

## Discussion

Having evolved over years with a continuous feedback loop between developers and users of both SRS3D and the Aquaria pre-release, the system gradually improved in many ways. In this paper, we describe the major design decisions that have been made with respect to the visualisations and the user interface to the Aquaria database. Some of our visual mappings - such as the colour scheme and the representation of secondary structures - have been prototyped and discussed with users prior to being implemented in the system. For the interaction with the 3D view, a user study has been conducted [24] that produced valuable results for the further development of Aquaria as well as other systems implementing gesture and voice control for molecular visualisation.

Many of the design decisions implemented in Aquaria could also be helpful for the visualisation of molecular dynamics. For example, the ability to focus on residues of a particular functional annotation and then inspect their range of motion within the structure could help illuminate their mechanism of action. Conversely, it would be useful to observe a particular mode of dynamics and then find the associated residues in the amino acid sequence. The tight integration of sequence and structure in the Aquaria user interface makes the required selection processes very easy.

## Conclusions

By employing a consistent visual vocabulary for data integrated from disparate sources, Aquaria provides a comprehensive experience for the user that invites exploration with a minimum of cognitive load.

As demonstrated in both case studies, the visual design of the user interface enables users to address the key questions identified in the requirements analysis (Table 1); these studies further show how addressing these questions leads to insight into the molecular mechanisms underlying protein function.

For future work, we would like to adapt Aquaria for mobile devices by implementing the entire user-interface in JavaScript. We are exploring the potential of newly available input devices -- such as depth- sensing cameras -- to simplify 3D control of molecular graphics [24]. We also aim at developing similar aggregation techniques for features as those suggested for sequences, render multiple protein structures in the 3D viewer and add further information visualisation techniques to aid in the analysis, such as parallel coordinates [25] to enable the analysis of spatially distributed attributes [26,27] or graphs to explore protein-protein interactions and pathways.

## List of abbreviations used

PDB: *protein data bank*, NGS: *next generation sequencing*, SNP: *single-nucleotide polymorphism*, PTM: *post-translational modification*, PMP: *protein model portal*, APP: *amyloid precursor protein*, 3D: *three-dimensional*, ePMV: *embedded python molecular viewer*

## Competing interests

The authors declare that they have no competing interests.

## Authors' contributions

CS led the interface design with contributions from SIOD. SIOD, AS, KSS and JH conducted the requirements analysis. The manuscript was written by CS, JH, SIOD, KSS and CJH. All figures were prepared by CS with contributions from SIOD and CJH, who also prepared the case studies. SIOD and AS conceived of the project, and participated in its design and coordination. All authors read and approved the final manuscript.

## Declarations

Publication of this work was supported by funds from CSIRO.

This article has been published as part of *BMC Bioinformatics *Volume 16 Supplement 11, 2015: Proceedings of the 5th Symposium on Biological Data Visualization: Part 1. The full contents of the supplement are available online at http://www.biomedcentral.com/bmcbioinformatics/supplements/16/S11
